# Non-alcoholic fatty liver disease and compromised endothelial function in people with type 2 diabetes

**DOI:** 10.1186/s12902-023-01460-w

**Published:** 2023-09-25

**Authors:** Zeinab Montazeri, Nahid Hashemi-Madani, Hamed Iraji, Masoudreza Sohrabi, Fariba Alaei-Shahmiri, Zahra Emami, Mohammad Reza Babaei, Mojtaba Malek, Mohammad E. Khamseh

**Affiliations:** 1 Endocrine Research Center, Institute of Endocrinology and Metabolism, Department of Internal Medicine, School of Medicine, Iran University of Medical Science, Tehran, Iran; 2 Endocrine Research Center, Institute of Endocrinology and Metabolism, Iran University of Medical Science, No. 10, Firoozeh St., Vali-asr Ave., Vali-asr Sq, Tehran, Iran; 3 Department of Interventional Radiology, Firouzgar Hospital, Iran University of Medical Science (IUMS), Tehran, Iran; 4 Gastrointestinal and liver diseases research center, Iran University of Medical Sciences, Tehran, Iran; 5 Research Center for Prevention of Cardiovascular Disease, Institute of Endocrinology and Metabolism, Iran University of Medical Sciences (IUMS), Tehran, Iran

**Keywords:** Type 2 diabetes, Non-alcoholic fatty liver disease, Flow-mediated dilation, Endothelial dysfunction, Cardiovascular disease

## Abstract

**Introduction:**

Nonalcoholic fatty liver disease (NAFLD) frequently coexists with type 2 diabetes mellitus (T2DM) and synergistically contributes to the development of atherosclerosis. Flow-mediated dilation (FMD) is a commonly used noninvasive test for assessing endothelial function. The main objective of this study was to explore FMD in patients with T2DM with and without NAFLD.

**Methods:**

In this cross-sectional study, conducted on people with T2DM, NAFLD was defined as controlled attenuation parameter (CAP) score > 302 dB/m. Endothelial dysfunction was detected when arterial FMD of brachial artery was equal or less than 0.7%. Regression analyses were applied to assess factors associated with impaired FMD.

**Result:**

A total of 147 patients (72 with NAFLD and 75 without NAFLD) were included in the final analysis. Patients with NAFLD were more likely to develop FMD ≤ 7% (77.8% vs. 58.7%, P = 0.01). In multivariate analysis, NAFLD (OR = 2.581, 95% CI (1.18–5.62), P = 0.017) and hypertension (HTN) (OR = 3.114, 95% CI (1.31–7.35), P = 0.010) were associated with an increased risk of impaired FMD. However, female sex was associated with a decreased risk of impaired FMD (OR = 0.371, 95% CI (0.15–0.87), P = 0.024).

**Conclusion:**

NAFLD is associated with endothelial dysfunction in people with T2DM. This risk is comparable with the risk imposed by HTN, highlighting the importance of screening and management of NAFLD in these patients.

## Introduction

Nonalcoholic fatty liver disease (NAFLD) is one of the most common liver diseases with a prevalence of about 25% in adults [1]. The prevalence is increasing worldwide due to the increasing rate of obesity and unhealthy lifestyle [2]. It might become the most common indication for the liver transplant [3]. Unhealthy lifestyle, obesity, dyslipidemia, and type 2 diabetes (T2DM) all can contribute to the development of NAFLD [4]. Moreover, sufficient evidence has been found that NAFLD is an early predictor and determinant of developing T2DM [5]. Multiple mechanisms have been proposed to contribute to the development of diabetes in patients with NAFLD among which insulin resistance plays the fundemental role [6]. An accumulation of free-fatty acids in the hepatocytes impairs post receptor signaling pathways ultimately results in hepatic insulin resistance [6]. Furthermore, over-activation of pathways involving pro-inflammatory cytokines, such as interlukin (IL)-1, IL-12, IL-18, and tumor necrosis factor-ᾳ, also contribute to insulin resistance [7]. Insulin resistance is a common pathological mechanism in both NAFLD and metabolic syndrome. Some studies considered NAFLD as a manifestation of metabolic syndrome associated with an increased risk of cardiovascular disease (CVD) [8, 9]. It contributes to the development of CVD via different pathways among which endothelial dysfunction occurs very early [10–12].

Various techniques are available to detect endothelial function. Fellow mediated dilation (FMD) is one of the most commonly used of these methods [13]. Many studies evaluated the association of NAFLD and FMD, suggesting the independent role of NAFLD in decreasing FMD [14]. However, these studies mainly focused on general population. Considering the coexistence of T2DM and NAFLD, we conducted this study to evaluate FMD in a population of diabetic patients with and without NAFLD.

## Methods

### Study population

Males and females with T2DM, aged between 30 and 75 years, and referred to the diabetic clinics at Institute of Endocrinology and Metabolism, Iran University of Medical Sciences, between 2019 and 2022. The exclusion criteria were as follows: (1) history of chronic liver disease of any etiology including viral and autoimmune hepatitis, (2) history of heart failure, (3) pregnant or lactating women, (4) using corticosteroids, (5) using medications causing hepatotoxicity, (6) alcohol intake more than 20 g/day in women or more than 30 g/day in men for at least 3 consecutive months during the last 5 years.

### Ethical approve

The study was carried out under the declaration of Helsinki and the International Conference on Harmonization of Good Clinical Practice (ICH-GCP) guidelines. The present study was approved by the IUMS Research Ethics Committee (REC.1399.1344). Informed consents were obtained from all participants prior to enrollment.

### Laboratory tests

The panel of requested laboratory tests included a complete blood count (CBC), fasting blood glucose (FBS), glycated hemoglobin (HbA1c), lipid profile, serum concentrations of alanine aminotransferase (ALT) and aspartate aminotransferase (AST), as well as serology for hepatitis B and hepatitis C. Additionally, antinuclear antibodies (ANA) test was requested to exclude autoimmune liver disease. All tests were done in fasting status. CBC was counted using sysmex K-21 device. HbA1C was measured using enzymatic method. FBS, AST, ALT, albumin, triglyceride, and cholesterol were measured applying photometric method. Solid-phase enzyme-linked immunosorbent assay (ELISA) was used for detection of hepatitis B surface antigen, hepatitis C anti-body, and ANA.

### FMD measurement

Diameter of the brachial artery measured using vascular probe placed 3–5 cm above medial epicondyle. Then a sphygmomanometer cuff was inflated for 5 min at a pressure of 200 mmHg or 50 mmHg higher than the arterial systolic pressure, to create distal limb ischemia. After deflating the cuff, the brachial artery diameter was re-measured again using ultrasound Doppler at the same location of the previous measurement. The measurement was done approximately 45 to 60 s after cuff deflation, to determine the anterior-posterior diameter of the brachial artery. For evaluation of FMD “philips affiniti 70 ultrasound and philips L12-3 linear probe” were used. We adopted the cut-off value for normal FMD from a previously published study in 2020. According to this study, FMD values greater than 7% were classified as normal, while FMD values equal to or less than 7% were considered abnormal [15].

### Controlled attenuation parameter (CAP) score measurement

According to a study conducted in 2019, we considered hepatic steatosis based on CAP score equal or greater than 302 dB/m [16].

### Statistical analysis

Statistical analyses were performed by SPSS Statistics for Windows (Version 25.0 IBM Corp. Released 2017. Armonk, NY). Continuous variables are expressed as mean ± standard deviation or as median (IQR) for skewed data. Categorical variables are presented as frequency (%). The study groups were compared using independent samples T-test, Mann-Whitney U test, or χ2 test, as appropriate. The associations of brachial FMD with demographic, clinical and laboratory risk factors were evaluated using multivariable logistic regression analyses with a backward variable selection method. All tests were 2-tailed, and the significance level was set at 0.05.

## Results

A total of 147 patients were included in the final analysis (45 females and 30 males with Non- NAFLD vs. 38 females and 34 males with NAFLD). Mean ± SD age for participants in patients without NAFLD and with NAFLD were 55.4 ± 9.4 and 55.8 ± 6.0 years, respectively. Baseline demographic and clinical information for both groups are presented in Table 1. In the NAFLD group, the body mass index (BMI) (30.1 vs. 27.5 kg/m2; p < 0.001), Triglyceride (TG) (165 vs. 126 mg/dl; P = 0.002), AST (20.5 vs. 19IU/l; P = 0.03), ALT (25 vs. 18 IU/l; P < 0.001), dyslipidemia prevalence (95.8 vs. 81.3%; P = 0.006), and CAP score (326 vs. 266Db/M; p < 0.001) were significantly higher than those without NAFLD. Moreover, the patients with NAFLD were more likely to develop FMD ≤ 7% (77.8% vs. 58.7%; p = 0.013).

**Table 1:** Characteristics of the study participants by NAFLD status

Variables	Non-NAFLD group (*n* = 75)	NAFLD group (*n* = 72)	P- value
Age (year)	55.4 ± 9.4	55.8 ± 6.0	0.80
Female, n (%)	45 (60.0%)	38 (52.8%)	0.38
BMI (kg/m^2^)	27.5 (25.6–30.0)	30.1 (27.9–33.0)	**< 0.001**
SBP (mmHg)	120.0 (110.0-125.0)	120.0 (110.0-130.0)	0.11
DBP (mmHg)	80.0 (80.0–80.0)	80.0 (80.0-83.5)	0.28
Fasting glucose (mg/dl)	141.0(109.5-166.5)	131.5(112.5-159.5)	0.57
HbA_1C_ (%)	7.5 ± 1.3	7.5 ± 1.5	0.88
TG (mg/dl)	126.0 (97.5-167.5)	165.0 (115.5-232.5)	**0.002**
Total chol. (mg/dl)	141.6 ± 38.2	148.8 ± 37.8	0.26
LDL-chol.(mg/dl)	70.1 ± 24.7	73.2 ± 23.8	0.59
HDL-chol. (mg/dl)	51.0 (43.5–59.5)	48.0 (40.0–54.0)	**0.01**
ALT (IU/l)	18.0 (13.5–23.5)	25.0 (19.0-30.5)	**< 0.001**
AST (IU/l)	19.0 (16.0-23.5)	20.5(18.0–26.0)	**0.03**
eGFR ( mL/min/1.73m^2^)	72.0 ± 13.2	72.7 ± 4.9	0.76
Current smokers, n (%)	7 (9.3%)	11 (15.3%)	0.27
Diabetes duration (year)	9.0 (3.5–14.5)	7.0 (4.5–12.0)	0.88
Insulin, n (%)	20 (26.7%)	16 (22.2%)	0.53
Anti-hypertensive, n (%)	32 (42.7%)	32 (44.4%)	0.83
Statin, n (%)	55 (73.3%)	53 (73.6%)	0.97
Hypertension, n (%)	32 (42.7%)	32 (44.4%)	0.27
Dyslipidemia, n (%)	61 (81.3%)	69 (95.8%)	**0.006**
CAP score (dB/m)	266.0 (245.0-280.0)	326.0 (316.0-340.0)	**< 0.001**
Brachial FMD ≤ 7%, n (%)	44 (58.7%)	56 (77.8%)	**0.013**
Paradoxical vasoconstriction (%)	6(8%)	9 (12.5%)	0.37

Continuous variables are expressed as mean ± SD or as median (IQR) for skewed data. Categorical variables are presented as n (% within group)

Based on the analysis for CAP Score quadrants (Table 2), the mean of BMI (P-value < 0.001), SBP (P-value = 0.005), TG (P-value = 0.001), total cholesterol (P -value = 0.03), and ALT (P-value = 0.002) significantly differ among the quadrants. However, there was no difference in the mean of FMD among the groups.

**Table 2:** Demographic and clinical characteristics of participants by CAP Score quartiles

Variables	CAP Score (dB/m)				P-value Between group
	Q1 [180.0-265.0, n = 37]	Q2 [266.0-300.0, n = 38]	Q3 [301.0-326.0, n = 36]	Q4 [327.0-400.0, n = 36]	
Age (year)	56.84 ± 8.6	54.05 ± 10.0	56.86 ± 6.2	54.67 ± 5.6	0.29
BMI (kg/m2)	26.40 (24.60–28.40)	28.85 (26.50–30.50)	28.75 (27.50–31.10)	31.35 (29.00-33.65)	**< 0.001**
SBP (mmHg)	120.0 (110.0-125.5)	120.0 (110.0-120.0)	120.0 (110.0-127.0)	126.5 (119.0-132.5)	**0.005**
DBP (mmHg)	80.0 (80.0–80.0)	80.0 (80.0–80.0)	80.0 (76.0–80.0)	80.0 (80.0–90.0)	0.07
FBG (mg/dl)	141.0 (115.0-161.0)	139.5 (108.0-171.0)	130.0 (109.0-160.5)	132.5 (115.0-157.0)	0.95
HbA1C (%)	7.4 (6.3–8.5)	7.4 (6.8-8.0)	7.1 (6.2–7.9)	7.2 (6.8–8.5)	0.22
TG (mg/dl)	109.0 (85.0-141.0)	143.0 (117.0-186.0)	151.5 (113.0-210.0)	182.0 (118.5–236.0)	**0.001**
Total chol. (mg/dl)	127.0 (107.0-169.0)	143.0 (116.0-177.0)	134.0 (114.5-160.5)	155.5 (131.5-180.50)	**0.03**
LDL-chol.(mg/dl)	67.43 ± 23.22	74.45 ± 25.84	68.61 ± 22.40	77.72 ± 24.53	0.22
HDL-chol.(mg/dl)	51.0 (44.0–59.0)	51.5 (43.0–60.0)	49.0 (39.5–53.0)	44.5 (40.5–56.5)	0.08
ALT (IU/l)	18.0 (13.0–26.0)	18.5 (15.0–23.0)	23.0 (17.5–29.0)	26.5 (20.0-31.5)	**0.002**
AST (IU/l)	19.0 (16.0–25.0)	18.0 (16.0–22.0)	19.0 (17.5–25.0)	22.0 (18.5–27.5)	0.08
eGFR (mL/min/1.73m^2^)	69.49 (61.34–77.75)	74.10 (67.95–80.20)	73.43 (60.08–84.47)	72.47 (64.48–80.81)	0.83
CAP Score (dB/m)	245.0 (235.0-260.0)	280.0 (270.0-290.0)	316.0 (310.0-320.0)	340.0 (330.0-360.0)	**< 0.001**
FMD Brachial (%)	5.00 (2.04–9.09)	6.35 (2.27–12.24)	3.89 (0.92-6.00)	4.49 (0.98–7.29)	0.11
Paradoxical vasoconstriction (%)	3 (8.1%)	3 (7.9%)	3 (8.3%)	6 (16.7%)	0.54

Continuous variables are expressed as mean ± SD or as median (IQR) for skewed data unless otherwise stated. Categorical variables are presented as n (% within quartiles)

### Association between FMD and clinical, and laboratory characteristics of the participants

The risk of impaired FMD defined as FMD ≤ 7% in people with NAFLD was 2.58 times more than that of non-NAFLD people (OR = 2.58, 95% CI (1.18–5.62), P-value = 0.017,) (Table 3). HTN was associated with an increased risk of FMD ≤ 7% (OR = 3.11, 95% CI (1.31–7.35), P-value = 0.010). However, risk of FMD ≤ 7% was significantly lower in women compared to that in men (OR = 0.37, 95%CI (0.15–0.87), P-value = 0.024).

**Table 3:** Evaluating the association between Brachial FMD and demographic, clinical and laboratory risk factors by logistic regression

Model	B	S.E.	P-value	OR (95% CI)
Gender, n (%)		0.438	**0.024**	0.371 (0.157–0.875)
Women	-0.991			
Men	Ref.			
Hypertension, n (%)		0.439	**0.010**	3.114 (1.318–7.356)
Yes	1.136			
No	Ref.			
Statin Medication, n (%)		0.439	0.069	2.226 (0.941–5.267)
Yes	0.800			
No	Ref.			
GFR, (mL/min/1.73m2)	-0.027	0.015	0.077	0.973 (0.944–1.003)
NAFLD Status, n (%)		0.397	**0.017**	2.581 (1.185–5.624)
NAFLD	0.948			
Non-NAFLD	Ref.			

**Dependent:** abnormal FMD defined as (FMD ≤ 7%)

**Independents:** Age; Sex; Smoking; Diabetes duration; BMI; SBP; DBP; HTN; DLP; Statin medication; Insulin medication; FBS; HbA1C; GFR; TG; Chol; HDL; LDL; AST; ALT; LSM; NAFLD

## Discussion

We found that endothelial dysfunction, defined as the impaired FMD, is more prevalent in people with T2DM and NAFLD compared to those with T2DM and without NAFLD (77.8% vs. 58.7%). Moreover, NAFLD was associated with 2.58 times increase in the risk of endothelial dysfunction.

NAFLD and CVD are complications of metabolic syndrome. Moreover, specific contribution of NAFLD to the occurrence of fatal and non-fatal CVD events has been previously reported [8, 9]. It seems that the association of NAFLD and CVD is independent of traditional cardiovascular risk factors, namely age, sex, BMI, waist circumference, smoking status, HTN, and dyslipidemia [9]. A more recent meta-analysis indicated synergistic impact of T2DM and NAFLD on the occurrence of CV events [17]. This study reported two times risk of CVD in patients with T2DM and NAFLD compared to those with T2DM and without NAFLD [17].

The mechanisms through which NAFLD contribute to the occurrence of CVD are very complex and involve different pathways. Genetics and epigenetics [18, 19], altered lipid metabolism, systemic inflammation [20], systemic insulin resistance [21], plaque formation [22], oxidative stress [23], altered gut microcbiome [24], and endothelial dysfunction are proposed as the possible contributing mechanisms [10, 12]. Endothelial dysfunction is an early step in the pathogenesis of atherosclerosis [11]. Decrease in nitric oxide synthase (NOS), increase in asymmetric dimethylarginine (ADMA), elevated serum homocysteine, decrease in vascular tone, and increase in oxidative stress all contribute to the development of endothelial dysfunction [10, 12]. The increased level of ADMA, an endogenous antagonist of NOS, as well as hyperhomocysteinemia is observed in patients with NAFLD [25, 26]. Many studies explored the association of NAFLD with endothelial dysfunction, as a common pathophysiologic mechanism in CVD [27–31].

Our study showed that NAFLD independently increases the risk of endothelial dysfunction defined as altered FMD of brachial artery. It has been reported that FMD is significantly lower in patients with NAFLD compared to those without NAFLD [28, 32, 33]. A recent meta-analysis of 5486 individuals demonstrated impaired FMD in patients with NAFLD [11]. However, it is worth noting the previous studies were conducted in the general population. Some previous studies showed the synergistic increase in CVD risk in patients with T2DM and NAFLD [17], however, none of them evaluated FMD in this particular population. In our study patients with T2DM and NAFLD had significantly higher risk of impaired FMD compared to those with T2DM but without NAFLD. The risk was comparable to the risk of impaired FMD imposed by HTN. Some study evaluated the relationship between FMD and cardiovascular risk factors demonstrating FMD adversely correlated with traditional cardiovascular risk factors including diabetes. Additionally, age, sex, and BP were strong independent predictors of FMD [34]. In the current study of a population of patients with T2DM the additive impact of NAFLD on FMD was independent of all possible confounders including age, sex, smoking status, duration of diabetes, BMI, SBP, DBP, DLP, use of statin, use of insulin, FBS, HbA1C, TG, Cholestrol, HDL, and LDL.

### Strengths and limitations

This study evaluated the risk of endothelial dysfunction in people with T2DM and NAFLD. All possible confounding factors that impair endothelial function were considered in the analysis. However, this was a cross-sectional study precluding us to draw causal inferences.

## Conclusion

The adverse effect of NAFLD on FMD comparable to the impact of HTN on FMD in people with T2DM highlights the importance of screening, detection, and management of NAFLD in this population.

## Data Availability

The datasets used and/or analyzed during the current study available from the corresponding author on reasonable.

## Abbreviations

ADMA: Asymmetric dimethylarginine
ALT: Alanine aminotransferase
ANA: antinuclear antibodies
AST: Aspartate aminotransferase
BMI: Body Mass Index
CAP: Controlled attenuation parameter
CBC: complete blood count
CVD: Cardiovascular disease
ELIZA: enzyme-linked immunosorbent assay
FBS: fasting blood glucose
FMD: Flow-mediated dilation
HTN: Hypertension
ICH-GCP: International Conference on Harmonization of Good Clinical Practice
NAFLD: Nonalcoholic fatty liver disease
T2DM: Type 2 diabetes mellitus
TG: Triglyceride
